# Adherence to labor and delivery care quality standards and associated factors among nurse-midwives in two public teaching and referral hospitals in Kenya: a cross-sectional survey

**DOI:** 10.11604/pamj.2023.45.149.36943

**Published:** 2023-08-04

**Authors:** Domisiano Koome Impwii, Lucy Kivuti-Bitok, Anna Karani

**Affiliations:** 1 School of Nursing and Public Health, Chuka University, Chuka, Kenya,; 2 School of Nursing Sciences, University of Nairobi, Nairobi, Kenya

**Keywords:** Adherence, standards, labor, delivery, nurse-midwives

## Abstract

**Introduction:**

maternal mortality is a major health concern, especially in low and middle-income countries. In Kenya, about 362 maternal deaths occur in every 100,000 live births. Seventy-five percent of these deaths can be prevented through the provision of quality care, especially during labor and delivery as per the quality standards. The objective of this study was to establish the level of adherence to labor and delivery care quality standards among nurse-midwives, and the factors hindering the adherence.

**Methods:**

a descriptive, cross-sectional survey was carried out in the maternity units of Embu and Meru Teaching and Referral hospitals in Kenya. A total of 51 Nurse-midwives were involved in the study. Data on adherence was collected through direct observation using an observation checklist, whereas that of factors hindering adherence was collected through face-to-face interviews using a semi-structured questionnaire. Data were checked, coded, and entered into EPI Info version 7.1.2. SPSS Version 25.0 was used to analyze data. Associations between variables were tested using Pearson correlation and Fisher's exact tests at 95% CI.

**Results:**

most of the participants (60.7%, n=31) were diploma holders, and a half (51%, n=26) were aged 20-29 years. About half (51%, n=26) had practiced for between 1 and 9 years and 43.1% (n=22) had worked in the maternity unit for more than a year. Out of the 12 quality standards assessed, only 5 (41.7%) were adhered to. Major implementation challenges include unavailability of standards (n=98.0%, n=50), inadequate supplies (96.1%, n=49), inadequate knowledge (88.2%, n=45), and an overwhelming workload (86.3%, n=44). There is a significant correlation between the highest level of qualification and lack of knowledge of quality standards (r=-0.279, p=0.05).

**Conclusion:**

adherence to labor and delivery care quality standards is low among nurse-midwives. Stakeholders must allocate more resources for training and the provision of adequate supplies. The facilities should also source for and customize the quality standards to promote greater adherence.

## Introduction

Maternal mortality remains a major health concern bedeviling many countries globally with about 295,000 mothers dying due to pregnancy-related complications each year [1]. In sub-Saharan Africa, it is estimated that 196,000 maternal deaths occur annually accounting for 66% of the global maternal deaths [1]. In Kenya, the current maternal mortality ratio (MMR) is 362 deaths per 100,000 live births which is way above the global level of 211 [2]. Hemorrhage, hypertensive disorders, and sepsis remain the most common direct causes of maternal mortality, especially in sub-Saharan Africa [3,4]. In Kenya, obstetric hemorrhage and hypertensive disorders account for most of the direct causes of maternal deaths [5-7]. These occur mainly during labor, delivery, or in the immediate postpartum period. Due to this fact, it has been argued that most maternal deaths can be reduced if mothers are delivered under the care of medically trained and licensed healthcare personnel. To this end, many countries in Africa, Kenya included, have either waived or decreased delivery fees in public health facilities to promote their use by mothers In Kenya, the waiving of delivery fees saw public health facility-based deliveries increase by 26.8% [8-10].

However, studies have shown that increasing accessibility does not significantly reduce the National MMR due to the poor quality of maternal health care [3, 11-13]. A confidential inquiry into maternal deaths carried out in 2017 in Kenya revealed that about 50% of maternal deaths were a result of substandard care and 92.4% received sub-optimal care during labor and delivery [7]. From the foregoing, it can be deduced that to reduce maternal mortality it is fundamental that quality of care is addressed [9]. Maternal health quality of care standards such as those developed by WHO (2016) are required to be utilized by health facilities to measure and continually improve quality of care [9]. Twelve of these standards relate to evidence-based practices for routine care and seven relate to the management of complications which have a greater impact in drastically reducing MMR if adhered to [9].

Adhering to quality of care standards improves quality of maternal health care provided. This in turn has the capacity of reducing maternal mortality by three quarters. Unfortunately, there are very few documented studies, if any, on the extent of adherence to quality of care standards by health facilities. Due to this, the quality of maternal health care being provided in the health facility cannot be stated with certainty. Therefore, the findings of this study will provide much needed information on the extent to which the health facilities are adhering to maternal health quality of care standards and any challenges that could be hindering the adherence. This will help the policy makers and program supervisors to come up with strategies to improve adherence, which would eventually improve quality of care and reduce maternal mortality.

**Objectives:** this study was carried out to 1) evaluate the extent to which labor and delivery care quality standards are adhered to, and 2) the challenges hindering compliance. Specifically, the study sought to determine the level of adherence on performance standards related to history taking and examination of mother in labor, appropriate care of mother during labor as well as assisting the mother have a clean and safe delivery. Additionally, the study sought to find out the level of adherence to standards on active management of third stage of labor, management of mothers in the immediate post partum period as well as instrument processing and waste management after delivery. This information is useful to all the stakeholders involved in ensuring that high-quality care is provided to mothers during labor, delivery and the immediate postpartum period thus reducing the risk of maternal mortality.

## Methods

**Study design:** a descriptive cross-sectional survey was carried out in Embu and Meru Teaching and Referral hospitals in Embu and Meru County respectively between December 2021 and March 2022.

**Study setting:** the study targeted nurse-midwives working in the maternity units of the Embu and Meru level five teaching and referral hospitals. Embu hospital is the largest referral hospital in the eastern south region serving Embu county and the surrounding counties of Tharaka Nithi, Kitui and parts of Machakos and Kirinyaga. It is located in Embu County at Latitude: 0° 31' 52.03" N and Longitude: 37° 27' 2.20" E coordinates. It has a 166-bed capacity maternity unit. Approximately 3,100 deliveries and 1,200 caeserian sections are conducted annually. In 2022, seven maternal deaths and forty two fresh still births occurred. Meru hospital is the largest referral hospital in the Eastern North region. It serves Meru county and the surrounding counties of Isiolo, Marsabit and Samburu. It is located in Meru County within Meru Municipality at latitude 0° 02' 46.54" N and Longitude: 37° 39' 21.13" E coordinates. It has a 128 bed capacity maternity unit. Approximately 3,300 deliveries and 1,700caeserian sections are conducted annually. In 2022, six maternal deaths and thirty-four fresh still births occurred.

**Participants:** the study involved nurse-midwives specifically in the antenatal and labor wards. Only nurse midwives who had worked in the unit for a period of six months or more and who consented were included in the study.

**Variables:** adherence to labor and delivery care standards refer to the extent to which the nurse midwives followed the laid down procedure during admission and management of the mother during admission, labor, delivery, and the immediate postpartum period. Each standard has specific actions referred to as the verification criteria (Table 1). Challenges refer to factors that prevent the nurse-midwife from carrying out an action as specified in the verification criteria or make the nurse-midwife carry out the action incorrectly. The potential confounders were the age and experience of the midwives. Training on basic and comprehensive essential obstetric care was considered to be an effect modifier.

**Table 1:** an example of a standard with verification criteria

Performance standards	Verification criteria	Yes, No, Not Applicable	Comments
9. The provider adequately performs active management of the third stage of labor	Observe if the nurse-midwife: palpates the mother's abdomen to rule out the presence of a second baby (without stimulating contractions)	/	/
	Tells the woman that she will receive an injection of oxytocin	/	/
	Administers 10 IU of oxytocin IM within 1 minute of birth	/	/
	Performs controlled cord traction	/	/
	After the expulsion of the placenta, massages the uterus with one hand on a clean/sterile cloth over the abdomen, until the uterus contracts firmly	/	/
	Examines the placenta and membranes to check if complete	/	/
	Measures blood loss; if the woman's condition is affected by the blood loss, decides immediate action	/	/

**Data sources/measurements:** data on adherence to care standards were collected through direct observation of midwives through an observation checklist as they admitted and cared for the mother during labor, delivery, and the immediate postpartum period. Data on challenges hindering the use of standards was collected through face-to-face interviews using a semi-structured questionnaire. The observation checklist was adapted from an assessment tool for measuring quality using the Standard based management -Recognition (SBM-R) quality improvement strategy by the ministry of health -Tanzania, 2011. The questionnaire was developed by the authors. The validity of the study instruments was tested by using carefully selected experts who examined the contents of the study instruments to determine whether the test was valid. A test-re-test method was used to ensure reliability, where the participants completed the same instruments at two different times. This method was used since the characteristic that was being measured does not change over time. The correlation coefficient(r) was then calculated, and a score of 0.82 was obtained, which is within the > 0.70-0.90 range, which is considered high. To avoid contamination the study instruments were tested in Chuka level 4 hospital and adjustments were made based on the findings of the pretest.

**Bias:** Hawthorne effect could influence nurse-midwives practice. To control this, nurse midwives were not informed of the specific variables being observed. Additionally, the participants were observed first and interviewed later.

**Study size:** a census was used to select the participants from the two health facilities. The sample size comprised 56 nurse-midwives, of which 29 and 27 were from Embu and Meru teaching and Referral hospitals respectively.

**Quantitative variables:** a standard is considered either adhered to or not. A standard is considered adhered to if all the nurse midwives observed carries out correctly all the actions as indicated in the verification criteria.

**Data analysis:** data collected were checked for completeness and consistency at the end of each day. Analysis of the data was done using Statistical Package for Social Scientists (SPSS) version 25.0 Descriptive statistics were used to present participants' characteristics, nurse-midwives compliance with standards guidelines as well as the compliance challenges. Pearson's correlation and Fisher's exact tests were used to test the relationships between variables at 95%CI. Both tests were selected since the data was quantitative. Fisher's exact test was used as it is recommended for testing relationship between quantitative variables when the sample is small. Pearson's correlation was used in order to determine the strength of relationship between variables. The relationship between compliance with the standards, socio-demographic characteristics, and barriers to compliance were tested.

**Ethical considerations:** ethical clearance to carry out the study was obtained from KNH/UON Ethics and Research Committee vide license no. KNH-ERC/A/468. Authorization to conduct the study at the two hospitals was given by the Medical Superintendent following perusal and approval of the research proposal by the research committee of the facility. Recruitment of the study participants was voluntary as no coercion or inducement was used. Informed and written consent was obtained from each participant after explaining the purpose of the study to each participant. Confidentiality of the information obtained was maintained by ensuring that no personal identifier is indicated in the questionnaire.

## Results

**Participants:** a total of 51 nurse midwives participated in the study out of 56 sampled, translating into a 91.1% response rate. Out of these, 52.9% (n=27) and 47.1% (n=24) were from Embu and Meru teaching and referral hospitals respectively. Out of those sampled, two nurse-midwives from Embu and two from Meru Hospital did not participate because they were on annual leave. Additionally, one nurse-midwife from Meru did not participate since she was on long sick leave during the period of study.

**Characteristics of participants:** most of the participants, 90.2% (n=46) were female and 60.7% (n=31) were diploma holders. Half of the participants (51%, n=26) were aged between 20 and 29 years. Regarding the experience of the respondents, the majority of the respondents, 51% (n=26) had practiced nursing for 1-9 years and 43.1% (n=22) had worked in the maternity unit for between six months and 1 year (Table 2). There is a positive correlation between experience and duration in the maternity unit (r=0.303, p=0.05).

**Table 2:** demographic characteristics of the participants

Variable	Category	Frequency	Percentage	Cumulative percentage
**Gender**	Male	5	9.8	9.8
	Female	46	90.2	100.0
**Age**	20 - 29 years	26	51.0	51.0
	30 - 39 years	16	31.4	82.4
	40 - 49 years	6	11.7	94.1
	50 years and above	3	5.9	100.0
**Marital status**	Never married	22	43.1	43.1
	Married	27	53.0	96.1
	Divorced/separated/widowed	2	3.9	100.0
**Highest academic qualification**	Certificate	1	2.0	2.0
	Diploma	31	60.7	62.7
	Degree and above	19	37.3	100.0
**Length of practice**	Less than 1 year	14	27.4	27.4
	1 - 9 years	26	51.0	78.4
	10 - 19 years	5	9.8	88.2
	20 - 29 years	6	11.8	100.0
**Duration in maternity**	<6months	13	25.5	25.5
	6months-1 year	16	31.4	56.9
	>1 year	22	43.1	100.0

**Nurse-midwives adherence to standards of care during labor and delivery:** the study sought to determine the extent to which the nurse-midwives were implementing the standards. Each of the nurse mid-wife admitted, assessed, and managed a mother in labor from the first stage of labor up to the immediate postpartum period. An observation checklist was used to assess whether the stipulated action is carried out and if it is carried out correctly. A total of 12 performance standards were observed out of which only 41.7% (n=5) were adhered to (Table 3). There was no correlation between nurse-midwives demographic characteristics and adherence to the standards of care.

**Table 3:** nurse-midwives' adherence to standards of care during labor and delivery

Standard of care	Verification criteria achieved	Verification criteria not achieved	Standard of care adhered to? (Yes, No, NA)
**The nurse midwife**: undertake a quick general assessment of the expectant mother in labor to identify signs of complications to prioritize care	9	2	N
Properly gathers and correctly documents the clinical history of the mother in labor	10	0	Y
Properly carries out physical examination between contractions	4	3	N
Properly conducts the obstetric examination	6	0	Y
Properly conducts a vaginal examination	8	0	Y
Plans and implements appropriate care during labor based on the history and physical examination results/findings	4	2	N
Uses the partograph to monitor labor and make adjustments to care	3	4	N
Assists the mother to have a clean and safe delivery	8	3	N
Correctly carries out active management of the third stage of labor	7	0	Y
Adequately performs immediate postpartum care	6	2	N
Properly clears the used instruments and disposes of medical waste after delivery	4	4	Y
Closely monitors the mother for at least two hours after delivery	0	5	N
Total number of performance standard			5(40.7%)

**Challenges hindering adherence to standards of care during labor and delivery by nurse-midwives:** the study sought to determine the challenges preventing nurse-midwives from complying with standards of care during labor and delivery. All the respondents, 100% (n=51) indicated that they were facing compliance challenges. The participants were then given several challenges and were asked to indicate whether they were important, undecided, or not important. Challenges indicated as important by a majority of the respondents include the unavailability of quality standards (98.0%, n=50) and inadequate supplies (96.1%, n=49). Other challenges include overwhelming workload (86.3%, n=44), lack of knowledge of standards (88.2%, n=45), and lack of incentives (82%, n=41) (Table 4). There is a significant correlation between the highest level of qualification and lack of knowledge of quality standards (r=-0.279, p=0.05).

**Table 4:** challenges hindering adherence to standards of care by nurse-midwives during labor and delivery

Variable	Category	Frequency	Percentage	Cumulative percentage
Standards not available	Important	50	98.0	98.0
	Undecided	0	0	98.0
	Not important	1	2.0	100.0
Inadequate supplies	Important	49	96.1	96.1
	Undecided	0	0	96.1
	Not important	2	3.9	100.0
Overwhelming workload	Important	44	86.3	86.3
	Undecided	2	3.9	90.2
	Not important	5	9.8	100.0
Inadequate knowledge of standards	Important	45	88.3	88.3
	Undecided	4	7.8	96.1
	Not important	2	3.9	100.0
Lack of incentives	Important	37	72.5	72.5
	Undecided	5	9.8	82.3
	Not important	9	17.3	100.0
Lack of management support	Important	6	11.8	11.8
	Undecided	30	58.8	70.6
	Not important	15	29.4	100.0

## Discussion

**Demographic characteristics:** most of the participants in this study were females aged between 20-29 years and had practiced for between 1-9 years. This could be attributed to the common practice in the deployment of nurse-midwives in most hospitals in Kenya, where younger nurses tend to be deployed in the maternity department. These findings disagree with others that assert that most of the nurses in Kenya are aged 31-40 years and 40-49 years respectively [10,11]. A majority (60.8%) of the nurse-midwives in this study were diploma holders. This could be explained by the fact that Kenya has more diploma training institutions and most of the certificate nurses have undertaken an upgrading program as directed by the Nursing Council of Kenya (NCK). This concurs with findings in other studies that most of the nurses in Kenya are diploma holders [10,11].

**Adherence to standards:** out of the 12 standards of care observed, only 41.7% (n= 5) were adhered to. Low scores in adherence to performance standards have been reported in other countries. In Afghanistan and Ethiopia for instance, before the introduction of a Standards-based management program, the adherence to standards was 26% and 28% respectively [12]. However, this contradicts findings from studies in Brazil where higher compliance scores were attained [13].

**Challenges hindering adherence:** lack of standards of care was an important compliance challenge, according to 98% (n=50) of the respondents. This was confirmed during the observation since the nurse-midwives could not produce a copy of the quality of care standards and guidelines. A copy of the standards acts as a resource for the nurse midwife. In its absence, the nurse-midwives may not be aware of what is expected of them. These findings agree with the findings by Babiker *et al*. and Fischer *et al*. that the absence of quality standards is a major impediment to their use [14,15].

Most of the participants, 96.1% (n=49) also cited inadequate supplies as an important challenge which was also noted during the observation. Some actions were not carried out or were carried out incorrectly because of a lack of essential supplies. These findings concur with those by Fischer *et al*., Wamalwa, and Zheng *et al*. [14,16,17]. Inadequate supplies may be due to inadequate funding by the national government since the management of hospitals was transferred to the county government following the promulgation of the 2010 Constitution of Kenya [8,18].

A majority of the respondents (86.3%, n=44) reported that they are unable to adhere to the standards because of the overwhelming workload. This may be attributable to severe staff shortages. This was noted during the study period where in many instances there were only two nurse-midwives per shift. This finding is identical to that of Wamalwa [16]. Staff shortage may be explained by high staff turnover due to natural attrition and the out-migration of nurses to European countries coupled with a moratorium on employment [10].

Inadequate knowledge of the standards was also reported by a majority of nurse-midwives (88.2%, n=45). This was also noted during the observation where some nurse-midwives were not aware of some of the actions that needed to be undertaken especially during the management of labor. This may be due lack of training and updates attributable to inadequate resources for training by the county and hospital management coupled with the withdrawal of donors who carried out most of the in-service training. Furthermore, there was no evidence that the ministry of health adapted and disseminated the WHO maternal health care standards at the health facility level. These findings agree with the findings in other studies [14,15,19]. This study enables the stakeholders to understand the quality of care being provided and the challenges thereof. This will enable them to come up with measures to address the challenges to improve quality. This study also has limitations that should be noted. Firstly, only a small sample was involved from only two health facilities all of which are public. Expanding the study to involve more facilities and also a comparison with private health facilities could be advantageous. Causation also cannot be established since the study utilized a cross-sectional approach.

## Conclusion

Adherence to the standards of care during labor and delivery amongst nurse-midwives is low. This is attributable to compliance challenges including lack of the standards, inadequate supplies, inadequate knowledge of the standards, and overwhelming workload. It is recommended that stakeholders need to allocate more resources for in-service training or update, and the provision of adequate supplies. The facilities should also source for and customize the quality standards to promote greater adherence. Further studies need to be carried out on the knowledge and skills level of the nurse-midwives in the management of obstetric emergencies especially postpartum hemorrhage and pregnancy-induced hypertension.

### What is known about this topic

- *The maternal mortality ratio is high in Kenya;*
- *Quality maternal care especially during labor, delivery, and immediate post-natal period can reduce maternal deaths by about 75%;*
- *Quality can be improved if health care providers adhere to standards of care*.

### What this study adds

- *Adherence to standards of care during labor and delivery by nurse-midwives is low;*
- *Nurse-midwives are facing major compliance challenges such as the unavailability of the standards, inadequate knowledge about the standards coupled with inadequate supplies, and an overwhelming workload that hinders adherence*.
